# Evidence for Gain Reduction by a Precursor in an On-Frequency Forward Masking Paradigm

**DOI:** 10.3813/AAA.919229

**Published:** 2018

**Authors:** Elizabeth A. Strickland, William B. Salloom, Erica L. Hegland

**Affiliations:** Department of Speech, Language, and Hearing Sciences, Purdue Univ, West Lafayette, Indiana, United States.

## Abstract

A forward masking technique was used to measure cochlear gain reduction which might be consistent with the medial olivocochlear reflex (MOCR). A 4-kHz signal was set at 20 dB SL, and an on-frequency forward masker adjusted to just mask the signal. Adding a pink noise precursor before the signal and masker **increased** the level of the masker needed to mask the signal, in contrast to what would be expected from theories such as additivity of masking. The magnitude and pattern of this increase was similar to the increase in signal threshold seen with an off-frequency masker following a precursor.

## 1. Introduction

It has long been known that a signal in a simultaneous masker may be detected at a lower signal-to-masker ratio following preceding sound, either an extension of the masker or a separate precursor [1]. This improvement in signal-to-masker ratio, also known as “overshoot”, has been linked to the active process in the cochlea [2, 3, 4], and may be a benefit of a decrease in cochlear gain, as might be produced by the medial olivocochlear reflex (MOCR). Due to possible confounding effects of suppression, more recently a forward masking paradigm has been used in which a short masker and signal are used to probe the auditory system, and then a preceding sound is added which is intended to evoke gain reduction. In these experiments, the lower leg of the growth of masking (GOM) function measured with an off-frequency masker shifts to higher levels, e.g. [5, 6, 7], or the psychophysical tuning curve (PTC) broadens, particularly on the low-frequency side, while the tip is unaffected [8, 9]. Studies of the time-course and duration of this effect support the idea that it is consistent with the time course of the MOCR [10, 11]. One drawback of using forward masking is that the effects produced by the precursor do not show the potential benefits of gain reduction, that is, the threshold signal-to-masker ratio is increased, in contrast to the decrease seen in overshoot. Also, while studies have used careful controls to show that the results are consistent with gain reduction rather than temporal integration of the precursor and masker (also called additivity of masking), the question always remains whether at least some of the effect might be due to additivity of masking or some other type of “forward masking”.

In running a control condition for another study, an on-frequency forward masking condition was found in which the threshold signal-to-masker ratio decreased following a precursor. This is not consistent with additivity of masking, and is similar to the improvement in signal-to-masker ratio seen in overshoot. In the present experiment, this on-frequency masking phenomenon is explored along with results from conditions that have been used previously to estimate gain reduction. It is argued that both sets of results may be consistent with gain reduction.

## 2. Methods

### 2.1. Subjects

Five listeners were tested, three males and two females. The age range was from 20 to 29 years, with a median of 21. Each subject was tested on a clinical battery, and had hearing thresholds within the normal range (≤ 15 dB HL) for octave frequencies from 250–8000 Hz, normal middle ear function (type A tympanograms), and present DPOAEs. The right ear of each subject was tested.

### 2.2. Stimuli

The signal was a 4-kHz sinusoid, with a duration of 10 ms including 5-ms cos^2^ onset and offset ramps (no steady-state). The masker was 20 ms including 5-ms cos^2^ onset and offset ramps. The signal and masker durations were chosen to be short enough that gain reduction by the MOCR, which has an onset and offset delay of approximately 25 ms [12, 13], would not be elicited in time to affect the signal. The precursor was a pink noise 50 ms in duration including 5-ms cos^2^ onset and offset ramps. This duration was chosen because previous psychoacoustic experiments have shown it is more effective than longer or shorter durations for eliciting gain reduction [11].

### 2.3. Procedures

For all experiments, a three-interval forced-choice task was used with a two-down, one-up (or two-up, one-down) stepping rule. This stepping rule targets 71 percent correct on the psychometric function [14]. An adaptive tracking program was used, with a step size of 5 dB for the first three reversals, and 2 dB for the remaining reversals. Each track had 12 reversals, with the last 8 averaged for threshold. Tracks with standard deviations larger than 5 dB were not included. The last two runs were averaged for each threshold shown.

Testing was performed in a double-walled sound-attenuating booth. Stimuli were generated in custom MATLAB (2012a, MathWorks, Natick, MA) software [15] with a Lynx II XLR sound card. Stimuli were passed through a headphone buffer (TDT HB6) to ER-2 insert earphones.

First, threshold was measured for the signal alone. Signal thresholds were 16, 16, 21, 20, and 16 dB SPL for S1 through S5 respectively. The signal was then set at 20 dB above quiet threshold (dB SL). This signal level was chosen from a pilot experiment, in which the effects explored in the current study were unexpectedly discovered. The level of an on-frequency masker was then adjusted until it just masked the signal. A precursor was then presented before the signal and masker, and masker level again adjusted to just mask the signal. The precursor level was constant for a run. Multiple precursor levels were tested for each subject to observe the effect of precursor level, which had already been measured in the second condition.

A second condition had been run previously for three of the subjects (S1, S2, and S3) as part of another study, and was run after the first condition for S5. S4 was not available for the second condition. The signal was set at 5 dB SL, and threshold found for an on-frequency (4-kHz) and off-frequency (2.4 kHz) masker that would just mask the signal. This signal level was chosen so that it should be on the lower leg of the GOM function, and thus should show the greatest magnitude of gain reduction. A precursor was then presented before the masker and signal. In the previous experiment, the masker level was set, and the signal level adjusted. Results from that experiment will be compared to the results of the current experiment.

## 3. Results

**Figure 1.**
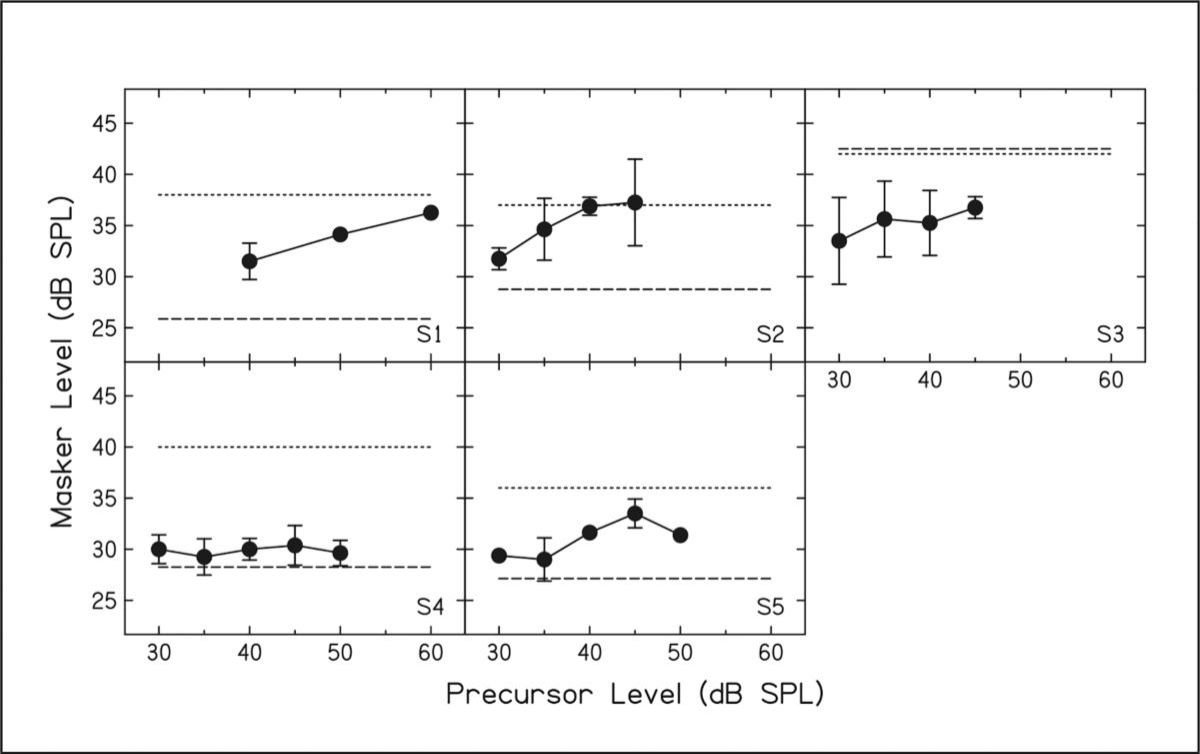
Masker thresholds needed to mask a signal set at 20 dB SL. Signal level is shown by the dotted line. Masker thresholds with no precursor are shown by the dashed line. Masker thresholds with a precursor are shown by the symbols.

The results for the first experiment are shown in Figure 1. The signal level (20 dB SL) is shown by the dotted line in each panel. The on-frequency masker level needed to mask the signal is shown as the dashed line in each panel. The threshold masker levels needed when a precursor is added before the signal and masker are shown by the symbols. Note that for four of the listeners (S1, S2, S4, and S5) the masker level increases following a precursor. This means that the signal-to-masker ratio (difference between symbols and dotted line) decreases following the precursor. Note that for S3, both the signal and masker lines are at the top of the panel, and masker threshold decreases with a precursor. However, the masker threshold does increase slightly with precursor level.

These results are intriguing, because they are in the opposite direction of what would be expected from a theory of additivity of masking. In order to further test the idea that the results are consistent with gain reduction, the results above may be compared to results from the second experiment. In that experiment, the signal was set at 5 dB SL. Maskers were on-frequency (4 kHz) or off-frequency (2.4 kHz), and were adjusted to find the level that would just mask the 5-dB SL signal. The off-frequency masker was chosen to be almost an octave below the signal frequency. As in previous experiments, it was reasoned that this masker frequency would have a linear growth of response at the signal frequency place, and would not be affected by gain reduction. If a precursor decreases gain at the signal frequency place, the increase in signal threshold should give an estimate of the amount of gain reduction in dB.

**Figure 2.**
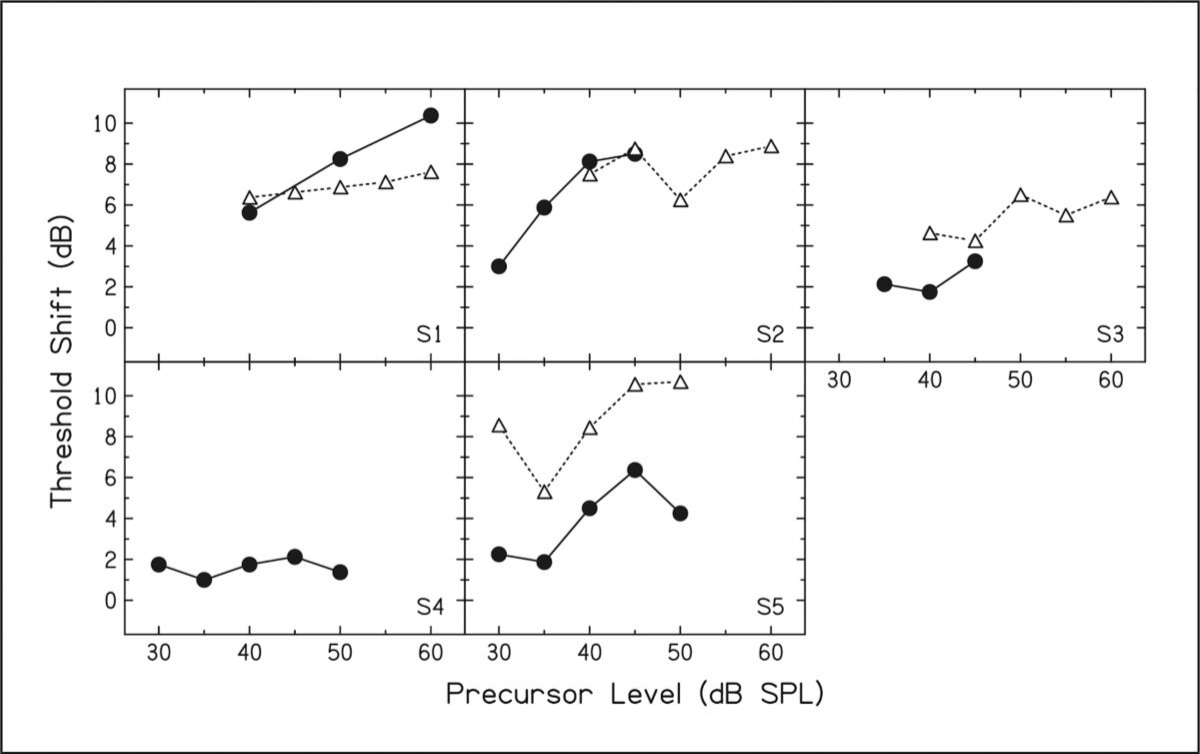
Threshold shift with a precursor compared across two methods. The filled circles show the difference in on-frequency masker thresholds with and without a precursor and the open triangles show the difference in signal thresholds with an off-frequency masker with and without a precursor. For S3, the masker thresholds are shown relative to masker threshold with a 30-dB precursor.

In Figure 2, the change in signal threshold with a precursor (open triangles) is shown for five subjects. As noted in the Methods section, S4 did not have time to participate in the second experiment. The data from Figure 1 are also replotted as the change in masker threshold with the precursor (filled circles). Note that in most cases the magnitude of the masker shift was equal to or less than the magnitude of the signal shift. Also note that the pattern of shift with precursor level is similar in cases where precursor levels used in the two conditions overlap.

As part of the second experiment, the effect of a precursor on signal threshold with an on-frequency masker was also measured. This was only done with a 60-dB SPL precursor. In this condition, the precursor increased signal threshold by at most 2 dB.

## 4. Discussion

These experiments use a forward masking paradigm that was developed in our laboratory, but has been used by other researchers to examine gain reduction such as might be produced by the MOCR [5,6,7]. In most previous studies, the differential effect of a precursor on signal threshold following an off-frequency masker vs. an on-frequency masker has been used to demonstrate effects consistent with gain reduction. In those studies, either signal level has increased or masker level has decreased which could also be consistent with additivity of masking between the precursor and masker. These studies have required the use of an off-frequency masker which must be assumed to produce a linear response at the signal frequency place. The off-frequency masker levels need to be rather intense to be able to mask the signal. Many studies have also used fairly high precursor levels.

In the present study, the results with a signal set at 20 dB SL show that the effects of a precursor may be demonstrated using an on-frequency masker, eliminating the need for a linear off-frequency reference. The effect of the precursor is to decrease the signal-to-masker ratio at threshold (shown by the level of the masker increasing), which cannot be modeled with additivity of masking. It is also important to note that the on-frequency masker effect was not seen with a lower signal level, arguing against alternative hypotheses such as the precursor making the masker inaudible. The on-frequency condition also allows the effect of a precursor to be seen at precursor levels well below those where the middle ear muscle reflex might play a role.

**Figure 3.**
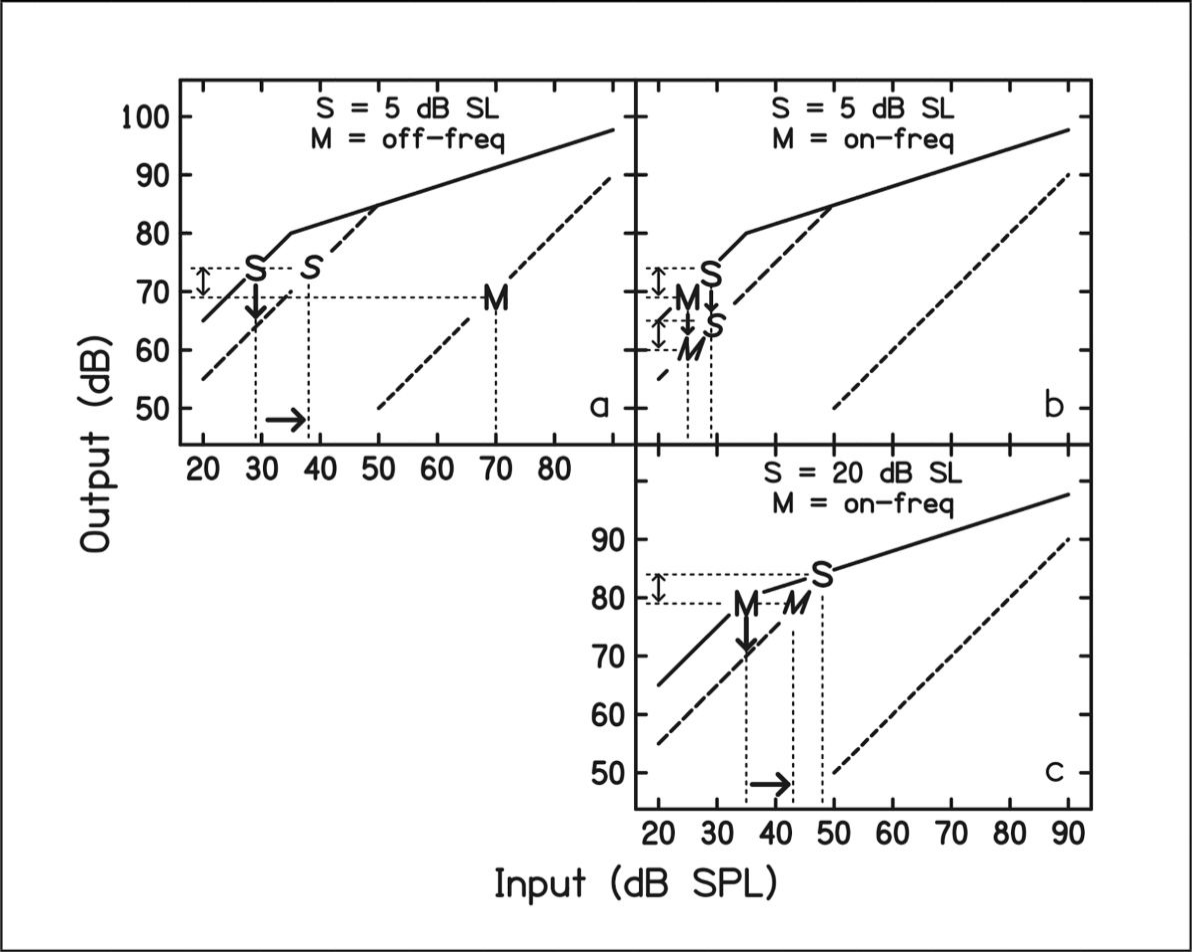
Schematic showing the effects of gain reduction on signal (S) and masker (M) levels. See text for details.

Figure 3 is a schematic representation of how gain reduction may produce the results seen in the present study. It is assumed that the listener attends to the output of the filter centered at the signal frequency (4 kHz). The inputoutput (I/O) function for the signal frequency is shown by the solid line, and approximates growth of excitation curves measured physiologically on the basilar membrane. Panel a shows a condition for a signal at 5 dB SL (represented by the S) and an off-frequency masker (M), conditions from the second experiment. The masker is approximately an octave below the signal frequency, and thus should have a linear I/O function and be unaffected by gain reduction. It is assumed that the listener can detect the signal at some constant signal-to-masker ratio, which would be the difference between the signal and masker levels at the output, shown by the double-headed arrow between the output levels for the signal and masker. If a precursor decreases the gain, shown as the dashed line, the signal will be affected (shown by the downward arrow), but the masker will not. Since the signal output decreases, the input signal level must be increased to achieve the original threshold signal-to-masker ratio, shown by the arrow to the right and the italicized S. In panel b, for a signal at 5 dB SL and an on-frequency masker, gain reduction may still occur with a precursor, but it affects the signal and masker equally, and thus there is no change in threshold. These two panels match the results from the second experiment.

Panel c of Figure 3 shows what may be happening in the first experiment, where the signal is set at 20 dB SL, and the masker is on-frequency. Gain reduction by the precursor reduces the masker output level, but not the signal output level. Therefore the masker level can be increased to achieve the criterion signal-to-masker ratio. The schematic shows the idealized condition, where the masker output is affected but the signal output is not. For the listeners in the present study, the underlying I/O function was not measured. However, for all listeners except for S3, the threshold signal-to-masker ratio for the on-frequency masker was higher when the signal was at 20 dB SL than when the signal was at 5 dB SL, which would be consistent with the levels shown in panels b and c. If the signal was affected by gain reduction as well as the masker, it would be predicted that the change in threshold following a precursor in the on-frequency condition would be less than that in the off-frequency condition, which is true in most cases. It is possible that for S3 the kneepoint of the I/O function is higher, and possibly also that the precursor caused a small amount of excitatory forward masking in addition to gain reduction. For S4, larger effects were seen in experiment 1 when the signal was set at 10 or 15 dB SL, consistent with the idea that the optimum signal level may depend on the individual I/O function.

The comparison to the results using a lower signal level is also consistent with the idea that the results reflect gain reduction. Although the signal is being adjusted in one case, and the masker in the other, Figure 3 shows that these stimuli should be on approximately the same part of the I/O function, and should both reflect effects of gain reduction. The fact that the magnitude and pattern of change in the stimulus following a precursor is similar across conditions supports the hypothesis that both effects may be due to the same mechanism.

The results of this study are also useful in that they reflect what may be one role of the MOCR in everyday life, which would be improving the signal-to-noise ratio under certain conditions. This has been demonstrated in a previous study using amplitude modulation (AM) [16]. That study found that detection of AM of a short, high- frequency carrier improved at mid carrier levels when a precursor preceded the carrier. However, AM detection was poorer at lower carrier levels (the lowest was 50 dB SPL) which the authors attributed to inaudibility. The present results allow the effects of gain reduction to be observed in an on-frequency task at lower stimulus levels, and thus extend the previous results.

The results of this study along with the results of previous studies are consistent with the hypothesis that the gain of the peripheral auditory system may shift by 10 dB or more in response to sound. It is important to consider the fact that the cochlear I/O function may be adjusting in response to sound when modeling effects beyond the cochlea.
